# Traffic Offloading in Unlicensed Spectrum for 5G Cellular Network: A Two-Layer Game Approach

**DOI:** 10.3390/e20020088

**Published:** 2018-01-28

**Authors:** Yan Li, Shaoyi Xu

**Affiliations:** School of Electronics and Information Engineering, Beijing Jiaotong University, Beijing 100044, China

**Keywords:** LAA, WiFi, Carrier Sensing and Adaptive Transmission, two-layer game, Collation-Auction Game-based Transaction, coexistence

## Abstract

Licensed Assisted Access (LAA) is considered one of the latest groundbreaking innovations to provide high performance in future 5G. Coexistence schemes such as Listen Before Talk (LBT) and Carrier Sensing and Adaptive Transmission (CSAT) have been proven to be good methods to share spectrums, and they are WiFi friendly. In this paper, a modified LBT-based CSAT scheme is proposed which can effectively reduce the collision at the moment when Long Term Evolution (LTE) starts to transmit data in CSAT mode. To make full use of the valuable spectrum resources, the throughput of both LAA and WiFi systems should be improved. Thus, a two-layer Coalition-Auction Game-based Transaction (CAGT) mechanism is proposed in this paper to optimize the performance of the two systems. In the first layer, a coalition among Access Points (APs) is built to balance the WiFi stations and maximize the WiFi throughput. The main idea of the devised coalition forming is to merge the light-loaded APs with heavy-loaded APs into a coalition; consequently, the data of the overloaded APs can be offloaded to the light-loaded APs. Next, an auction game between the LAA and WiFi systems is used to gain a win–win strategy, in which, LAA Base Station (BS) is the auctioneer and AP coalitions are bidders. Thus, the throughput of both systems are improved. Simulation results demonstrate that the proposed scheme in this paper can improve the performance of both two systems effectively.

## 1. Introduction

Global mobile data usage has grown by 70–200 percent per annum [1]. Long Term Evolution (LTE) has been proven to be a great success in satisfying the growing user data rate requirements owing to its high spectral efficiency, seamless coverage, and excellent centralized coordination and management services. However, the current capacity of LTE/LTE-Advanced networks will not be able to support the demand in the future. In recent years, enabling cellular small cells in unlicensed frequency bands has garnered attentions as a promising solution to the scarcity of the licensed spectrum for cellular networks [2,3]. The main incumbent system in the 5 GHz band is the Wireless Local Area Networks (WLAN) [4] which is designed in the premise of trading off performance for low cost and simple implementation for spectrum sharing [5]. The 3rd Generation Partnership Project (3GPP) proposed to extend the use of LTE to the unlicensed spectrum, which is named LTE in Unlicensed (LTE-U), also called License Assisted Access (LAA) [6]. Many articles focus on the fairness and friendly coexistence between LAA and WiFi, however, it is also critical to enhance the spectral efficiency of both two systems and make better use of the valuable spectrum resources.

Some coexistence mechanisms for LAA and WiFi have been studied in [7,8,9,10,11,12], which show that LTE and WiFi could coexist in a friendly manner, and improve the performance of the whole network. There are, at this stage, three main LTE mechanisms under considerations for ensuring fair coexistence with WiFi, namely, Listen Before Talk (LBT) [7,8], Carrier Sensing and Adaptive Transmission (CSAT) [9,10,11,12,13,14] and traffic offloading [15,16,17,18,19]. In the LBT mechanism, a transmitter would listen to the medium and determine whether it could transmit [6,7], which is similar to the incumbent Carrier Sense Multiple Access with Collision Avoidance (CSMA/CA) mechanism in IEEE 802.11 [5]. In practice, due to the occurrence of the collision, the LBT mechanism cannot clearly reflect the advantages of LTE, especially when too many users access to the channel. Furthermore, this method will reduce the whole networks throughput. To exert the advantages of LTE better, a LBT combined with CSAT (LBT-based CSAT) scheme is proposed in [9] which uses LBT to listen to the channel firstly and make sure the channel is free to enter, and then the CSAT could perform “on” for coexistence [10]. In addition, CSAT could be applied without changing the current LTE/LTE-Advanced technology, which adopts “on” and “off” state transition within a single duty cycle [11,12]. Moreover, CSAT could achieve the same fair effect as the LBT by adjusting parameters appropriately [13,14]. However, collision still may occur when the WiFi backoff time is counted down to zero and the LTE is about to send packets simultaneously at the start of “on” periods. To deal with the problem mentioned above, a modified LBT-based CSAT scheme, which increases a protection mechanism is proposed in this paper. In addition, data offloading is also a good way to share the spectrum. Offloading parts of data to the WiFi network [15] can effectively alleviate the LAA network congestion and enhance the user experience. The author in [16] proposed a new mechanism that is contrast to the traditional mobile data offloading, which transfers some WiFi users to be served by the LAA system. Meanwhile, some unlicensed spectrum resources may be allocated to the LAA system in compensation for handling more WiFi users. In this way, a win–win situation would be generated since LAA can generally achieve better performance than WiFi due to its capability of centralized co-ordination [4,20].

The IEEE 802.11 Working Group (which standardizes WiFi) is continuously developing new amendments to improve the WiFi performance in emerging scenarios [5]. However, some Access Points (APs) have overlapping coverage to share the network, which would greatly increase the number of devices competing for access. In addition, collisions in overlapping areas could lead to deterioration of the whole network’s throughput [21], as well as the additional energy consumption [22], especially for those APs which have too many stations. Conversely, few stations in one AP would mean the waste of AP resource, which cannot maximize the WiFi network performance. To address the issues mentioned above, we intend to make a balance on stations through associating the optimal station number in a WiFi AP and improve the channel resource utilization in a game way in this paper.

Game theory has an important position in wireless resource management and there are also some game theory applications in LAA field [17,18,19,23]. In [17], a spectrum sharing scheme based on the decision tree was built through the channel condition, load condition, etc., and then parts of data were offloaded from the operator which asked for a favor to the operator with few stations based on a repeated game. However, LTE and WiFi operators belong to different communication protocols and it is not practical to predict the state of the other operator and use the dynamic game with complete information in fact. A second-price reverse auction game was built in [18]: LAA network choses to work in a competition mode or in a cooperation mode according to their announced reserve rate. If they work in a competition mode, LAA randomly accesses the unlicensed channel and competes with WiFi based on a typical coexistence mechanism such as CSAT or LBT. For the cooperation mode, the LAA would carry the traffic of WiFi users in exchange for the exclusive access of the corresponding channel. Ref. [19] proposed a transaction from LTE-LAA to WiFi using CSAT way together with offloading, and then formulated a coalition game to improve resource utilization. In addition, in [23], a LAA-APs grand coalition was built and LAA could offload some data to WiFi networks when LAA traffic load is high within the grand coalition. However, the transmission efficiency of WiFi is much worse than LTE and the spectrum utilization of the WiFi network is bound to suffer from potential packet collisions due to its contention-based access protocol, especially when the number of competing WiFi users grows large, which would have bad effects on the whole network performance.

However, existing works on spectrum sharing or traffic offloading mostly concentrate on the performance optimization in LAA, but ignore the throughput improvement in WiFi. In addition, many existing works use a simple single-layer game between two systems, which cannot effectively solve the problems of both simultaneously optimizing the performance of two systems and the load balancing issue in WiFi system. In this paper, we propose a two-layer game, namely a Coalition-Auction Game-based Transaction (CAGT) game in the whole network in 5 GHz unlicensed bands. A coalition game is used among WiFi APs in the first layer to balance the traffic and reduce collisions. The key idea of the proposed coalition forming is to merge the light-loaded APs with heavy-loaded APs into a coalition, consequently, the data of the heavy-loaded APs can be transferred to the light-loaded APs such that the maximum WiFi throughput can be achieved. In the second layer, an auction game between the LAA base station and AP coalitions is performed to better utilize the spectrum resources and implement the friendly coexistence between two systems. In this game, LAA BS is the auctioneer and AP coalitions are bidders; parts of WiFi data can be offloaded to LAA network in compensation for allocating more unlicensed resources to the LTE system.

The main contributions of this paper can be summarized as follows.

- To avoid the collision at the start of “on” period [13] in the CSAT mechanism, a modified LBT-based CSAT structure is designed, which could effectively increase the throughput of both LAA and WiFi.
- To balance the stations among APs, the optimal station number that could maximize the average WiFi system throughput is obtained. The optimization problem is a fraction programming problem which means it is a NP-hard problem to be solved. Through analyses, the function is proved to be a quasi-concave function problem and it can be transferred to the subtractive form to deal.
- In addition, to increase the throughput of both LAA and WiFi, a CAGT mechanism by using LBT-based CSAT and traffic offloading is devised. The CAGT mechanism is a two-layer game. In the first layer of the game, a coalition game is built to make a load balance among WiFi APs, and then, in the second layer of the game, a second-price sealed-bid auction between LAA and WiFi heterogeneous systems is used to optimize the throughput of the whole network.

The remainder of the paper is organized as follows. Section 2 describes the system model. The optimization problem about optimal station numbers is also presented in this section. Section 3 shows the basic and the modified LBT-based CSAT structure. The proposed two-layer CAGT is described in Section 4 and Section 5. Simulation results are presented in Section 6, and the paper is finally concluded in Section 7.

## 2. System Model

### 2.1. The System Model of the Whole Network

**Figure 1:**
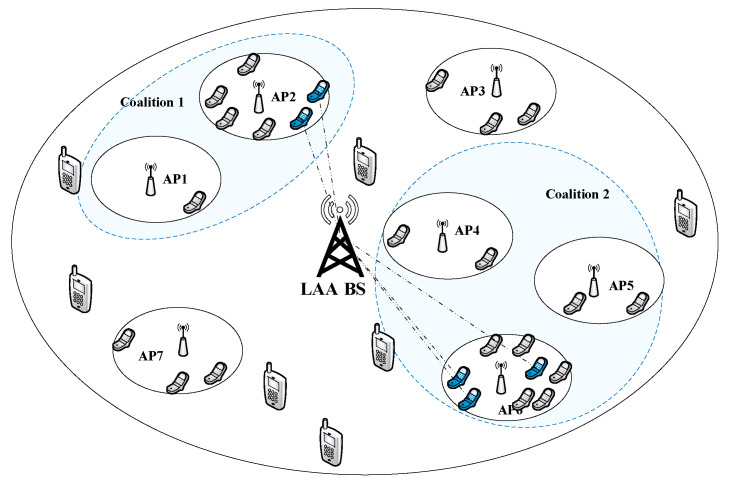
System Model.

As illustrated in Figure 1, we consider a coexisting network of one LTE-LAA macrocell and K={1,…,K} WiFi APs. These APs are overlaid on the coverage of the macrocell. Both the macrocell and the APs share the same unlicensed spectrum around 5 GHz and there is no interference between the macrocell and the APs due to the use of CSAT mechanism. We assume that WiFi users are also equipped with LTE transceivers and the number of WiFi users in the *k*-th AP is $N_{k}$. To facilitate the coexistence of WiFi and LAA, we further assume that there exists an inter-system coordinator, which performs the WiFi user transfer and unlicensed resource allocation, as in [24]. APs in a coalition can transfer stations from each other and enable devices to exchange information and perform actions without human intervention [22]. To implement this mechanism, a modified frame structure is used and managed by a centralized controller [25]. In addition, the APs may share the same channel with other internal/external APs and each AP covers a certain service area which may overlap with the service area of other APs. Each station can be associated only with one single AP at a time. The traffic arrival rate of a user is assumed to be known and the users have the same arrival rate. Furthermore, the time period in the unlicensed spectrum will be divided into several time slots, some for WiFi and the others for LAA. This can be accomplished by the CSAT method in [13,14] as controlled by the inter-system coordinator. In addition, the ratio of “on” to “off” can be adjusted adaptively to meet the needs of the different operators’ traffic load.

### 2.2. System Performance Analysis

We consider a scenario with one LAA Base Station (BS) and *K* APs coexisting in unlicensed band. The throughput of LTE in unlicensed spectrum can be expressed as

(1) $$C=B_{LAA}\log_{2}\left(1+SINR\right)\frac{T_{on}}{T_{on}+T_{off}}.$$

where $B_{LAA}$ is the bandwidth of LAA, SINR is the Signal to Interference and Noise Ratio, $T_{on}$ and $T_{off}$ mean the time for LAA “on” and ”off” periods, respectively.

According to [21] and the principle of the CSAT scheme, the per-user saturation throughput of the WiFi network can be expressed as

(2) $$R=\frac{P_{tr}P_{S}E\left[P\right]}{\left(1-P_{tr}T_{\sigma }\right)+P_{tr}P_{S}T_{S}+P_{tr}\left(1-P_{S}\right)T_{c}}\frac{T_{off}}{T_{on}+T_{off}},$$

where $T_{S}$ means the average time the channel is detected occupied because successful transmission occurs, $T_{c}$ denotes the average time the channel is sensed busy by each station during a collision, $T_{\sigma }$ is the duration of an idle slot time and E[P] represents the average packet size.

Let $P_{tr}$ and $P_{S}$ be the probability that there is at least one transmission in the considered slot time and the probability of successful transmission, respectively; they can be expressed as

(3) $$P_{tr}=1-{\left(1-\tau \right)}^{n},$$

and

(4) $$P_{S}=\frac{n\tau {\left(1-\tau \right)}^{n}}{P_{tr}}=\frac{n\tau {\left(1-\tau \right)}^{n-1}}{1-{\left(1-\tau \right)}^{n}},$$

where *n* is the stations number contending on the channel and each station transmits with probability τ. τ only depends on the network size and on the system parameters of maximum backoff stage and the size of the contention window.

However, too many stations will increase the collision probability and decrease the capacity [23]. Conversely, too few stations will not make full use of the channel spectrum resource. Thus, the number of stations in one AP should be carefully determined to maximize the capacity of the WiFi system. To best utilize the spectrum resources and maximize the system capacity, a coalition game among WiFi APs in the first layer of our CAGT optimization scheme is designed, and our goal is to optimize the station number $n^{*}$ in one AP coalition to maximize the throughput of the WiFi system. Accordingly, we reshape (2) and the optimization problem can be formulated as

(5) $$\underset{n\in N^{+}}{max}R=\frac{an\tau {\left(1-\tau \right)}^{n-1}E\left[P\right]}{\left(T_{\sigma }-T_{c}\right){\left(1-\tau \right)}^{n}+\left(T_{S}-T_{c}\right)n\tau {\left(1-\tau \right)}^{n-1}+T_{c}},$$

where $a=\frac{T_{off}}{T_{on}+T_{off}}$. Define $f\left(n\right)=an\tau {\left(1-\tau \right)}^{n-1}E\left[P\right]$ and $g\left(n\right)=\left(T_{\sigma }-T_{c}\right){\left(1-\tau \right)}^{n}+\left(T_{S}-T_{c}\right)n\tau {\left(1-\tau \right)}^{n-1}+T_{c}$. Thus, Equation (5) can be expressed as $R=\frac{f(n)}{g(n)}=\frac{-f(n)}{-g(n)}$, where −f(n) is concave and −g(n) is convex, which are judged by the Hessian Matrix. Then, according to [26], the function *R* is quasi-concave. Thus, the optimization problem can be expressed as

(6) $$\underset{n\in N^{+}}{\eta (n)}=\underset{n\in N^{+}}{max}R=\frac{f(n)}{g(n)}.$$

As mentioned in [27], any optimization problem in fractional form can be transformed into an equivalent optimization problem in a subtractive form. Hence, the non-linear fractional optimization problem in Equation (6) can be transformed into a parameterized function and the optimal solution can be determined by finding the root to the U(n) as shown in Equation (7) using various root finding methods [28,29],

(7) $$U\left(n\right)=\underset{n\in max\left\{\left\lfloor R^{+}\right\rfloor ,\left\lceil R^{+}\right\rceil \right\}}{max}\left\{-f(n)-\eta (-g(n))\right\}=\underset{n\in \left\{\left\lfloor R^{+}\right\rfloor ,\left\lceil R^{+}\right\rceil \right\}}{max}\left\{-f(n)+\eta g(n)\right\}.$$

The solution to Equation (7) could be formulated as an iterative two-layer solution combining Dinkelbach type method and Lagrangian dual decomposition approach. In addition, this problem can be solved by mathematical tools such as CVX or other build-in functions in MATLAB [29].

Due to concavity of the function analyzed above, balancing the stations and making the station number as close as the optimal value $n^{*}$ could improve the performance of the whole WiFi system. Therefore, we form a coalition game by encouraging individuals in the WiFi system to form groups in the first layer of CAGT mechanism, and then make a traffic transfer between LAA and WiFi in the second layer of CAGT.

## 3. Modified CSAT Mechanism

**Figure 2:**
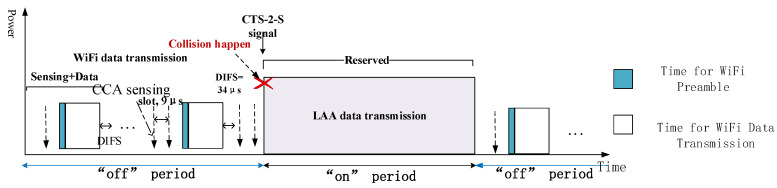
Incumbent LBT-based CSAT frame structure.

Before discussing the CAGT scheme, the basic and modified CSAT will be devised firstly. The primary principle of CSAT is to let LTE in unlicensed bands cycle between state “on” and “off” within a given single cycle while the LTE in licensed band remains connected all the time. During the activated state “on”, LTE transmits data on the unlicensed band and blocks the transmission of WiFi systems. However, within the deactivated state “off”, LAA shuts down its transmission to yield the channel sources to WiFi [11], just as shown in Figure 2. Adjusting “on” and “off” time dynamically by identifying the number of WiFi terminals can be an affordable way for proper “on” and “off” time assignment and meet better needs between system aggressiveness and fairness [12]. However, WiFi nodes may not be aware of the LAA activity in some actual scenarios. For example, WiFi causes interference to LTE in the “on” state period since WiFi cannot identify the structure of signals from non-IEEE systems. When the Energy Detection for Clear Channel Assessment (CCA-ED) measurement is below the predefined inter Radio Access Technology (inter-RAT) threshold, the ongoing LAA messages cannot be identified and will be regarded as noises. Therefore, a LBT based CSAT was proposed by Qualcom to deal with the above problem by which LAA should sense the channel (like the LBT) firstly before the cycle period turn “on” and ensure that there is no WiFi under transmission [9,10]. In addition, a Clear To Send to Self (CTS-2-S) message [8,9] can be inserted to reserve the channel resources for LTE transmission during the next “on” state period. Figure 2 illustrates the function of CTS2S message. The duration of upcoming “on” state is included in the CTS-2-S message. If WiFi cannot identify the ongoing LAA signals below the inter-RAT threshold, the corresponding WiFi backoff mechanism cannot be triggered in “on” period. While after introducing the CTS-2-S signal in LAA, the neighboring WiFi will back off until the LTE completes its transmission during the reserved “on” period. Therefore, the possible interference between these two systems is avoided within the time of LAA transmission. In this way, WiFi detect the channel to be busy and collision can be alleviated effectively. However, collision may still occur when the WiFi backoff time is counted down to zero and the LTE is about to send packets simultaneously at the moment of starting “on” periods, just as shown in Figure 2.

**Figure 3:**
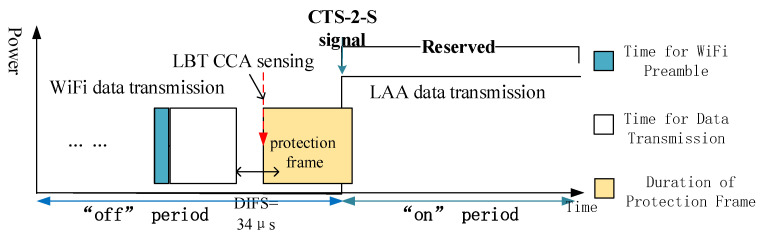
Modified LBT-based CSAT frame structure.

In terms of WiFi CCA sensing process, the AP which wants to transmit data will sense the channel for every 9 μs slot continuously until it counts down to zero and occupies the channel. In addition, a 34 μs Distributed Inter-Frame Spacing (DIFS) would follow the WiFi data for the general DCF structure. To some extend, the next WiFi message must wait for a 34 μs DIFS time and then wait until the station finishes the CCA sensing before it enters the channel as shown in Figure 2. To solve the collision problem mentioned above, a modified LBT based CSAT structure is proposed in Figure 3, where a special protection frame is inserted before the coming “on” period. ETSI 301 893 [30] specifies that before a transmission, a station must perform a CCA using energy detection for at least 20 μs. That means CCA sensing period is shorter than the DIFS time. Once the medium is sensed idle before the coming “on” period, LAA could occupy the channel before any WiFi station does. In addition, the protection frame we designed is a special almost blank frame with a fixed structure which could be recognized by LTE-LAA. Thus, the channel is reserved for LAA until the following “on” stage. By this way, the channel is sensed busy by WiFi before the “on” stage and collision would not happen anymore at the starting moment of “on” stage.

## 4. Coalition Game

In this section, we mainly consider a communication system with multiple APs on the “off” state period, because the CSAT method was adopted in the heterogeneous network. For the sake of simplicity, we only consider APs which access to different channels.

As mentioned above, because the light-loaded APs and heavy-loaded APs exist simultaneously, the maximum system throughput cannot be obtained. To solve this problem, the first layer of the two-layer CAGT is proposed. In the first layer, a coalition game is performed to balance the load among APs. The light-loaded APs and heavy-loaded APs have motivations to form a coalition such that the data of the heavy-loaded APs can be transferred to the light-loaded APs. To realize this mechanism, the APs periodically report the statistics regarding the station numbers and channel information to the centralized controller. In addition, the centralized controller periodically executes the proposed coalition forming algorithm using the reported information and notifies the results to all stations and APs. Depending on the association result, some stations may be required to perform handover to other APs and some APs may be turned on/off. Take the example in Figure 1, AP2 and AP6 are overloaded while AP1, AP4 and AP5 have only few users (stations), so none of them could gain the maximal throughput. If the coalition between AP1 and AP2 is formed, and another coalition among AP4, AP5 and AP6 is also formed, the user balance among APs could bring benefits to all of them. Next, we will describe the coalition game that heavy-loaded APs can transfer some stations to light-loaded APs to balance the stations.

### 4.1. Coalitional Game Definition

Define the distributed traffic offloading process as a coalitional transferable utility game (S,ν), where S denotes the set of players and ν(S) is the utility function of the coalition *S*. Note that, in our assumption, only the players with heavy traffic ask for a favor and the players which would like to help onload data are permitted to form a coalition together.

**Definition** **1.** *(Utility of the player) The utility of player i in a coalition* ν(i) *is defined as the difference after the player i joining the coalition* S:ν(i)=ν(S∪i)−ν(S).

At the beginning of each stage, players with their own stations compete for the channel based on their own transmitting needs respectively, according to its own station numbers, channel conditions and other competing AP numbers in the network. After that, players consult with the coalitionists to reach a consensus. To motivate players to onload data from APs with heavy load which ask for a favor, an encouragement factor α is put forward. We define $\alpha_{s}$ as the prize for giving a hand and a prize $\alpha_{a}$ for asking for a favor as follows:

(8) $$\left(\alpha_{s},\alpha_{a}\right)=\left\{\begin{array}{ccc} \alpha_{s}=-\beta R\left(n^{*}-n_{i}\right), & \alpha_{a}=0,\; & n_{i}<n^{*}\\ \alpha_{a}=\beta R\left(n_{i}-n^{*}\right), & \alpha_{s}=0,\; & n_{i}>n^{*} \end{array}\right.,$$

where β is a discount factor related to the sensitivity of giving a reward and $\beta \in \left[0,1\right]$. $n_{i}$ is the AP station number in stage *i*. The rule is that if the AP’s encourage factor α is negative and there are other APs which asks for a favor at the current stage, the AP has to help onload data. On the contrary, the coalition player whose encouragement factor is positive can offload data successfully. Players know rewards and rules in advance and the encourage factor for the operator is

(9) $$\alpha =\sum_{i}\left(\alpha_{s,i}+\alpha_{a,i}\right),$$

where *i* is the index of the stage and the centralized controller updates information periodically. The expected utility of one coalition is

(10) $${\bar{\nu }}_{coalition}=\sum_{i}\left[-\hat{P_{i}}\left(\alpha_{s,i}-\varepsilon_{i}\right)+\hat{Q_{i}}\left(\alpha_{a,i}-\varepsilon_{i}\right)\right],$$

where $\hat{P_{i}}$ and $\hat{Q_{i}}$ are the probabilities of offering help and asking help, respectively. $\varepsilon_{i}$ is the cost of investment and we assume that all players have the same $\varepsilon_{i}$. Thus, each player has the incentive to obtain the optimal stations in way of helping other APs carrying stations or asking for a favor to transfer some stations in order to increase its utility.

**Definition** **2.** *(Transfer rule of the coalition structure) Collecting all coalition structure possibilities to one set, namely* $Y=\{S_{1},S_{2},\ldots ,S_{L}\}$, *L is the total number of possibilities that a coalition structure could form. For a given coalition structure* $S_{L}$ *and* $S_{L^{\prime }}$, *if* $\nu \left(S_{L}\right)\leq \nu \left(S_{L^{\prime }}\right)$, *then coalition structure* $S_{L^{\prime }}$ *is better than* $S_{L}$. *The formula can be expressed as:*

(11) $$S_{L}\to S_{L^{\prime }}\Leftrightarrow \nu \left(S_{L}\right)\leq \nu \left(S_{L^{\prime }}\right),1\leq l^{\prime },l\leq L.$$

**Theorem** **1.** *The criterion for coalition formation to find proper players to minimize* $\sigma_{i}$ *is*

(12) $$-\sum_{i}n_{i,s}\hat{P_{i}}\alpha_{i,s}+\sigma_{i}=\sum_{i}n_{i,a}\hat{Q_{i}}\alpha_{i,a},$$

*where* $n_{i,a}$ *is the number of stations that needs to be transferred at the stage i, and* $n_{i,s}$ *is the number of stations which is promised to be onloaded by the APs which join in the coalition and have less traffic.* $\sigma_{i}\left(\sigma_{i}\geq 0\right)$ *is the correction factor which is related to the AP station number and the traffic in the whole channel.*

**Proof.**  It will be proven through the following two cases:

Case 1: $\sigma_{i}=0$. Once after the coalition algorithm is executed, $\sigma_{i}=0$ means the overloaded AP transfers parts of data successfully and just leaves $n^{*}$ stations. At the same time, the APs with less traffic can accept stations from the heavy-loaded APs. Hence the station number of each AP reaches to $n^{*}$ finally, namely, $\sum_{i}n_{i,s}P_{i}\alpha_{i,s}=\sum_{i}n_{i,a}Q_{i}\alpha_{i,a}$. Moreover, if several APs work in the network and all APs station numbers are less than $n^{*}$, some APs could be turned off and offload their data to other APs for transmitting, and finally every AP has $n^{*}$ stations. Thus, $\sigma_{i}=0$ is an ideal situation which means the station numbers in every AP are equal to $n^{*}$ after onloading and offloading, as well as the performance of the whole WiFi network could reach the optimum value in this case.

Case 2: $\sigma_{i}>0$. However, on some occasions, the ideal situation cannot be reached after searching the whole channel, so a correction factor σ is utilized to balance the stations. In this case, the AP which has too much traffic could get help from other APs in the same coalition to reduce its burden but cannot balance every AP station number equal to $n^{*}$. Due to the benefit for the players and the whole network’s performance improvement after coalition forming, the APs still tend to cooperate with the overloaded AP. Thus, our aim is to minimize $\sigma_{i}$ and make the active stations number close to $n^{*}$ as much as possible. ☐

### 4.2. Station Allocation Algorithm

Firstly, we use some notations to simplify our expressions: $n_{j,a}\left(j\in \left[1,K_{a}\right]\right)$ means the number of stations which will be transferred from the overloaded $AP_{j}$ and $n_{w,s}\left(w\in \left[1,K_{s}\right]\right)$ indicates the number of stations which could handover to the $AP_{w}$ (the AP which only has few stations). In addition, there are $K_{s}\left(K_{s}\in \left[1,K\right]\right)$ light-loaded APs and $K_{a}\left(K_{a}\in \left[1,K\right]\right)$ APs are overloaded ($K_{s}+K_{a}=K$). Then, the station allocation problem is shown in Algorithm 1 when there are many APs in the coalition and have the same channel condition and priority.

**Algorithm 1** Station Allocation Algorithm.
1:$\bar{w}$ indicates the subscript of the AP with lightest load and $\bar{j}$ denotes the serial number of the AP with the heaviest load in this coalition.2:**Initialization**: $\bar{w}=0$, $\bar{j}=0$.3:**loop**4: **while** $n_{w,s}<n^{*}$ **do**5:  $\bar{w}=min\left\{n_{1,s},n_{2,s},\ldots ,n_{w,s}\right\}$6:  **if** $K_{a}=0$ and $K_{s}>1$ **then**7:   **if** $\left\lceil \frac{\sum_{w}n_{w,s}}{n^{*}}\right\rceil <K_{S}$ **then**8:    transfer stations in $AP_{\left\lceil \frac{\sum_{w}n_{w,s}}{n^{*}}\right\rceil +1},\ldots ,AP_{S}$ to $AP_{1},\ldots ,AP_{\left\lceil \frac{\sum_{w}n_{w,s}}{n^{*}}\right\rceil }$9:   **else**10:    **if** $K_{a}>1,K_{s}>1$ and $\sum_{j}n_{j,a}>\sum_{w}n_{w,s}$ **then**11:     $\bar{j}=max\left\{n_{1,a},n_{2,a},\ldots ,n_{j,a}\right\}$12:    **end if**13:    transfer one station from $AP_{\bar{j}}$ to the $AP_{\bar{w}}$14:   **end if**15:   $n_{j,a}=n_{j,a}-1$16:   $n_{w,s}=n_{w,s}+1$17:  **end if**18: **end while**19:**end loop**20:Output station allocation results

**Theorem** **2.** *If* $K_{a}>1$, $K_{s}>1$ *and* $\sum_{j}n_{j,a}>\sum_{w}n_{w,s}$, $AP_{w}$ *would help the AP which has more stations preferentially and reduce the differences in each* $AP_{j}^{\prime }s$ *station number*.

**Proof.**  If $K_{a}>1$, $K_{s}>1$ and $\sum_{j}n_{j,a}>\sum_{w}n_{w,s}$, the throughput after coalition game would increase but cannot reach the optimal value. As we discussed in Section 2.2, the WiFi throughput function *R* is a quasi-concave function, which means when the station number is larger than $n^{*}$, the throughput would decrease with the station number *n*. To maximize the system performance, the station number in each AP should close to $n^{*}$ as much as possible. Thus, $AP_{k}$ should compare the station numbers every time when it onloads data and makes the final station number in every AP to an equal value as much as possible. ☐

**Theorem** **3.** *If* $K_{a}>1$, $K_{s}>1$ *and* $\sum_{j}n_{j,a}<\sum_{w}n_{w,s}$, $AP_{j}$ *would transfer its stations preferentially to the AP which has less stations and reduce the differences in each* $AP_{w}^{\prime }s$ *station number.*

**Proof.**  If there are both heavy-loaded APs and light-loaded APs in a coalition, when the station number is less than $n^{*}$, the throughput function is an increasing function of the station number *n*. Similar to the proof in Theorem 2, balancing the station numbers among $AP_{w}s$ is the first choice of optimizing system performance. ☐

### 4.3. Merge-and-Split Based Coalition Formation Algorithm

In this section, a distributed coalition formation algorithm is shown in Algorithm 2. We construct this algorithm based on rules denoted as “merge-and-split” which permits to modify a partition of *T*.

**Algorithm 2** Distributed Coalition Formation Algorithm.
1:Define *K* coalitions as $S=\left\{S_{1},S_{2},\ldots ,S_{K}\right\}$ in the network and every coalition has an AP at the start of the coalition game.2:**Initialization**: $\nu_{d}^{*}=0$, $S_{d}^{*}=\phi$3:**for** i,j∈S **do**4: **if** $\nu \left(S_{i}\right)\cup \nu \left(S_{j}\right)>\nu \left(S_{i}\right)$ **then**5:  $\nu \left(S_{i}\right)=\nu \left(S_{i}\right)\cup \nu \left(S_{j}\right)$, $S_{j}=\phi$6: **else**7:  **if** $\nu \left(S_{i}\right)\cup \nu \left(S_{j}\right)<\nu \left(S_{i}\right)$ **then**8:   $\nu \left(S_{i}\right)=\nu \left(S_{i}\right)\setminus \nu \left(S_{j}\right)$9:  **end if**10: **end if**11: **if** $\nu \left(S\right)>\nu^{*}$ **then**12:  $\nu_{d}^{*}=\nu \left(S\right)$13:  $S_{d}^{*}=S$14: **end if**15:**end for**


We define the merging rule as: For ν(i)≥0, if ν(S∪i)>ν(S), the player *i* would join in this coalition. In addition, we define the splitting rule as: For ν(i)≥0, if ν(S∖i)>ν(S), the player *i* would get away from this coalition. Three states in the coalition based on merge-and-split rule are shown when a new period begins or any player joins or leaves the coalition spontaneously.

State 1 Initial State. Each AP node is a single coalition and *K* coalitions are formed totally.
State 2 Merged State. The coalition would be formed among overloaded APs and light-loaded APs. The formed coalition could help each AP achieve the nearly optimal station number within its coverage area. AP coalitions could work either in the centralized schedule way or in the free competition way. After occupying the channel, data transmission could be allowed for APs in this coalition.
State 3 Split State. Splitting any coalition if the utility of the player after the split is larger than the utility before the split or if there are better association choices for the player, which means the coalition cannot exist steadily. A split form would happen in this condition before any new coalition is composed.

**Definition** **3.** *(The principle of a player joining a new coalition) Player* $i\in S_{1}$ *tends to leave its original coalition and to join* $S_{2}$ *if and only if* $\nu \left(S_{2}\cup \left\{i\right\}\right)≻\nu \left(S_{1}\right)$.

The algorithm starts at a partition where each AP itself is a coalition. Then, in each iteration, the AP will join in another coalition which can maximize the game value. Finally, until all APs form stable coalitions, the algorithm outputs the final partition. According to the repeated cyclical merging and splitting, every AP can find the optimal combination structure.

### 4.4. Computational Complexity Analysis

In general, the computational complexity is considered as the times that merge-and-split rule and transfer rule of coalition structure are used. The proposed scheme can effectively reduce the computational complexity because APs would form coalitions based on their station numbers and their own $\alpha_{s}$ and $\alpha_{a}$. We assume that there are $K_{s}$ light-loaded APs, so the number of heavy-loaded AP is $K-K_{s}$. In the beginning of the coalition game, each AP is a single coalition and *K* coalitions are formed totally.

Case 1: $K_{s}>K-K_{s}$. In this case, there are more light-loaded APs than heavy-loaded APs and we consider there is only one heavy-loaded AP in a coalition after the game. The light-loaded AP will join in the heavy-loaded AP coalition to help reduce its burden based on the merger rule. If there are better heavy-loaded coalition choices for this light-loaded AP, it will split from current coalition based on the spitting rule and join in the new coalition. For each light-loaded AP, it has $K-K_{s}$ choices, so the theoretical computational complexity of this algorithm is $\left(K-K_{s}\right)K_{s}$.
Case 2: $K_{s}\geq K-K_{s}$. In this case, there are more heavy-loaded APs. The heavy-loaded AP only could cooperate with APs which can help reduce its burden. Similarly, according to the merge-and-split rule and game prize, the theoretical computational complexity of this algorithm is $K_{s}\left(K-K_{s}\right)$.

In summary, the theoretical computational complexity of our proposed algorithm is $K_{s}\left(K-K_{s}\right)$.

### 4.5. Stability Analysis

As illustrated in Algorithm 2, an AP could either choose to join a new coalition or remain in its current coalition. To obtain the stable coalitional structure, a dynamic coalition formation model based on a Discrete Time Markov Chain (DTMC) can be formulated [31].

The state of the DTMC can be expressed as the set of all the coalition structures in Definition 2 denoted by $Y=\{S_{1},S_{2},\ldots ,S_{L}\}$, where *L* is the total number of coalitions formed and $S_{i}\cap S_{j}=\phi$.

The state space of the Markov chain is

(13) $$\Theta =\{\left(Y_{1}\right),\ldots ,\left(Y_{x}\right),\ldots ,\left(Y_{M}\right)\},$$

where $Y_{x}$ represents a coalition structure and *M* is the Bell number obtained from

$$M\left(i\right)=\sum_{j=0}^{i-1}\left(\begin{array}{c} i-1\\ \;j \end{array}\right)M\left(j\right),\;\forall i>1\;and\;M\left(0\right)=1.$$

The transition probability from one partition to another depends on the decisions of the players to leave and join the coalitions, which is denoted by a transfer matrix *P* and the elements of which are transition probabilities $\rho_{Y,Y^{\prime }}$,

(14) $$\rho_{Y,Y^{\prime }}=\left\{\begin{array}{cc} \prod_{r\in R}P_{x}\left(x\in S_{L}\in Y\to x\in S_{L}^{\prime }\in Y^{\prime }\right)\varphi_{i}\left(Y^{\prime }|Y\right), & \;Y\neq Y^{\prime }\\ 1-\Sigma_{i\in \Theta ,i\neq Y}\;\rho_{Y,i}, & \;Y=Y^{\prime } \end{array}\right.,$$

where $P_{x}\left(x\in S_{L}\in Y\to x\in S_{L}^{\prime }\in Y^{\prime }\right)$ is the transition probability the $AP_{x}$ changes from coalition $S_{L}\in Y$ to coalition $S_{L}^{\prime }\in Y^{\prime }$. The detailed derivation is given in Appendix A. In addition, $\varphi_{x}\left(Y^{\prime }|Y\right)$ is the probability that the player $AP_{x}$ decides to move from its current coalition $S_{L}$ to a new coalition $S_{L}^{\prime }$, which makes the coalitional structure to change from Y to $Y^{\prime }$, which can be expressed as:

(15) $$\varphi_{x}\left(Y^{\prime }|Y\right)=\left\{\begin{array}{cc} \vartheta , & \;\nu \left(S_{L}^{\prime }\right)\geq \nu \left(S_{L}\right)\\ 0, & \;otherwise \end{array}\right.,$$

where 0<ϑ≤1, $S_{L}\in Y$ and $S_{L}^{\prime }\in Y^{\prime }$.

The solution of the coalition formation game is the coalitional structure that can exhibit internal and external stability notions [32]. Internal stability implies that, given a coalition, no player in this coalition has an incentive to leave this coalition and obtain a higher payoff. External stability indicates that, given a partition, no player can improve its payoff by switching its current coalition and joining another one. In addition, the solution for the DTMC can be obtained in a centralized manner by using the inter-RAT centralized controller and the stable coalitional structure can be selected randomly by using the Algorithm 2 to find the solution based on the individual decision of each player. A stable coalitional structure can be identified from the stationary probability of the Markov chain defined with the state space in Equation (13) and transition probability in Equation (14). The stationary probability of the Markov chain can be obtained by solving

(16) $${\vec{\pi }}^{T}P={\vec{\pi }}^{T},$$

where ${\vec{\pi }}^{T}={\left[\pi_{Y_{1}},\ldots ,\pi_{Y_{M}}\right]}^{T}$, ${\vec{\pi }}^{T}\vec{1}={\vec{\pi }}^{T}$ is a vector of stationary probabilities and $\vec{1}$ is a vector of ones. $\pi_{Y}$ is the probability that a coalition structure is formed.

For the Markov chain defined by the state space and the transition probability, there could exist an ergodic set Ξ∈S, if $\rho_{Y,Y^{\prime }}=0$ for any Y∈Ξ and $Y^{\prime }\notin \Xi$, and no nonempty subset of Ξ has this property. A stable coalition structure will evolve if the formation process reaches a singleton ergodic set, namely, absorbing state $\rho_{Y,Y^{\prime }}=1$ for Y∈Ξ. At an absorbing state, there is no player that has an incentive to merge or split.

The transition probability matrix Q of the absorbing DTMC [31] can be partitioned as follows:

$$Q=\left[\begin{array}{cc} T & F\\ 0 & I \end{array}\right]$$

where T is the transition probability matrix corresponding to the transitions among the transient states, I is an identity matrix, 0 is a zero matrix and F is the transition probability matrix corresponding to the transitions from the transient states to the absorbing state. For an absorbing DTMC with transition probability matrix Q, the matrix $M={\left(I-T\right)}^{-1}$ is its fundamental matrix. The entry $m_{Y,Y^{\prime }}$ of M gives the expected number of times that the process is in transient state $Y^{\prime }$ if it starts in transient state Y before the Markov chain reaches any absorbing state. From the Theorem 1 of [33], if $\sum_{i\in S}\nu \left(\left\{i\right\}\right)<\nu \left(S\right)$, there is at least one absorbing state which is a stable solution of the coalition game. Since the payoff of a player will be zero if the player acts alone, the players would rather to be a member of coalitions to obtain a higher payoff if they have the chance. The ν(S) will be equal to or higher than zero, and then a stable solution may exist depending on the value of the payoff. Because the light-loaded AP can only form coalitions with the heavy-loaded AP, the stable coalition may exist when there are more than one players in a coalition. That is to say, a stable coalition may exist when there are both the light-loaded AP and the heavy-loaded AP in a coalition, namely, the utility of the coalition is larger than zero.

## 5. Auction Game

After making a balance among WiFi APs, the second layer of CAGT algorithm, an auction game is performed between LAA and WiFi coalitions to coexist more friendly and boost the spectrum utilization of two systems simultaneously. We assume that there are *L* coalitions totally after the coalition game. In the auction game, the role of the auctioneer is played by the LAA BS, while the AP-coalition plays the role of the bidder. A second-price sealed-bid auction, namely Vickrey auction in our work is applied. Firstly, LAA determines a base price $d_{i}=\omega_{i}\left(i=1,\ldots ,N\right)$, which means LAA would participate in the auction only when $d_{i}\geq \omega_{i}$, where $d_{i}$ is slots in which WiFi compensates for LAA in helping reduce the traffic burden and $w_{i}$ means the actual slots which were used in WiFi data transmitting by the LAA network. When the auction begins, AP coalitions have $m_{1},m_{2},\ldots ,m_{N}$ bits data waiting for transferring to the LAA network respectively. Next, LAA choose the AP coalition which could maximize the value of $\left(d_{i}-\omega_{i}\right)$ to cooperate. If $(d_{i}-\omega_{i})<0$, WiFi and LAA would continue working in their original way without offloading between two networks and the utility of players is zero.

For the second-price sealed-bid auction, the auctioneer selects the highest bidder, but only needs to pay the second high price to the bidder which wins the game. An advantage of this type of auction over others is that the dominant strategy for each bidder is to bid his true valuation. In addition, the game is repeated for *I* rounds and because the price is sealed, the information in auction progress cannot be known by their AP opponents. However, the bidders could adjust their prices and make better choices in the next auction round after the end of a round according to the result and their own needs. Interactive information could exchange among APs through a central controller. In the round i≤I, each agent submits a bid based on the value of the current unit, and then LAA BS offloads the traffic of the winner with the largest bid and charges the second bid. In addition, the AP coalition which has won the game can also join the later auction. Every AP coalition has the right to choose whether to participate in the auction at the beginning of each round of the auction. In this paper, we assume that, if the AP can gain benefits from the auction, it would take part in the auction.

### 5.1. Problem Formulation

We first investigate the benefit that the WiFi network can achieve after the auction. As mentioned before, the WiFi system achieves its benefit through transferring parts of users to the LAA system and reduce collisions. Thus, the average throughput of the AP coalition *i* after the auction can be expressed as

(17) $$R_{i}\left(N-\tilde{n}\right)=\frac{1}{L}\sum_{i=1}^{N-\tilde{n}}\frac{P_{tr}P_{S}E_{i}}{\left(1-P_{tr}T_{\sigma }\right)+P_{tr}P_{S}T_{S}+P_{tr}\left(1-P_{S}\right)T_{c}}\frac{T_{off}-d_{i}{\bar{T}}_{slot}}{T_{on}+T_{off}},$$

where $E_{i}$ is the mean number of bits sent by station *i* in a successful transmission [34], ${\bar{T}}_{slot}$ is the average time slot, *N* is the total number of stations competing for the channel in a AP coalition and there are $\tilde{n}$ stations transferred to the LAA network with $d_{i}$ relinquished slots.

The utility of the WiFi is the promoted throughput after the auction, that is

(18) $$\begin{array}{cc} u_{WiFi} & =\frac{1}{L}\sum_{i=1}^{N-\tilde{n}}\frac{P_{tr}P_{S}E_{i}}{\left(1-P_{tr}T_{\sigma }\right)+P_{tr}P_{S}T_{S}+P_{tr}\left(1-P_{S}\right)T_{c}}\frac{T_{off}-d_{i}{\bar{T}}_{slot}}{T_{on}+T_{off}}\\ & \;-\frac{1}{L}\sum_{i=1}^{N}\frac{P_{tr}P_{S}E_{i}}{\left(1-P_{tr}T_{\sigma }\right)+P_{tr}P_{S}T_{S}+P_{tr}\left(1-P_{S}\right)T_{c}}\frac{T_{off}}{T_{on}+T_{off}}\\ & =\frac{T_{off}-d_{i}{\bar{T}}_{slot}}{T_{off}}R_{i}\left(N-\tilde{n}\right)-R_{i}\left(N\right)\\ & =\frac{T_{off}-\theta }{T_{off}}R_{i}\left(N-\tilde{n}\right)-R_{i}\left(N\right), \end{array}$$

where $\theta =d_{i}{\bar{T}}_{slot}$ means the time which is compensated for LAA. The revenue of the WiFi comes from parts of data offloaded to LTE in unlicensed bands, which reduces the probability of sending packet and collisions in WiFi network. That is to say, the burden of AP can be reduced effectively.

The utility of the auctioneer is the throughput earned by LAA after the auction and it can be formulated as

(19) $$\begin{array}{cc} u_{LAA} & =r\frac{T_{off}}{T_{on}+T_{off}}+r\delta_{i}\left(d_{i}-\omega_{i}\right){\bar{T}}_{slot}-r\frac{T_{off}}{T_{on}+T_{off}}\\ & =r\delta_{i}\left(d_{i}-\omega_{i}\right){\bar{T}}_{slot}=r\delta_{i}\theta_{i}-\delta_{i}R\left(\tilde{n}\right), \end{array}$$

in the premise of $d_{i}\geq \omega_{i}$. Here, $\delta_{i}$ is the discount factor of LAA participating in game and $\delta_{i}\in \left[0,1\right]$. $\omega_{i}=\frac{R(\tilde{n})}{r{\bar{T}}_{slot}}$ means the number of time slots which is actually used in LAA by transmitting the WiFi data of $\tilde{n}$ stations.

### 5.2. A Second Price Auction Design

Next, a win–win strategy for both systems is discussed. The general win–win problem to balance the WiFi and LAA benefits can be expressed as a multi-objective function problem:

(20) $$max\;\left\{u_{WiFi},u_{LAA}\right\},$$

subject to

$$d_{i}\geq w_{i},$$

$$0\leq \tilde{n}\leq N.$$

Equation (20) is a multi-objective problem. Next, we will use the Nash equilibrium solution method [16,35] to solve the win–win strategy fairly.

**Definition** **4.** *(*$u_{WiFi}^{NES}$, $u_{LAA}^{NES}$*) is a Nash equilibrium solution if it solves the following problem*

(21) $$\left(u_{WiFi}^{NES},u_{LAA}^{NES}\right)=\underset{\tilde{n},d_{i}}{max}\;u_{LAA}u_{WiFi}.$$

*subject to* $d_{i}\geq w_{i}$ *and* $0\leq \tilde{n}\leq N$.

Therefore, solving the multi-objective optimization problem above is equivalent to solving

(22) $$\underset{\tilde{n},d_{i}}{max}\;u_{LAA}u_{WiFi}.$$

This problem can be solved by getting the second-order derivative of Equation (22) on $\theta_{i}$, that is

(23) $$\frac{\partial^{2}u_{LAA}u_{WiFi}}{\partial \theta_{i}^{2}}=\frac{2rR(N-\tilde{n})}{T_{off}}<0,$$

which means Equation (22) is a concave function and we can get the Nash Equilibrium solution through first-order derivative on $\theta_{i}$ as zero, as

(24) $$\theta_{i}^{*}=max\;\left\{\frac{T_{off}r\left[R\left(N-\tilde{n}\right)-R\left(N\right)\right]+R\left(N-\tilde{n}\right)R\left(N\right)}{2rR(N-\tilde{n})},\frac{R(\tilde{n})}{r}{\bar{T}}_{slot}\right\}.$$

In addition, the proposed LAA-WiFi auction process is described in Algorithm 3 and $I_{max}$ denotes the set of AP coalitions with the maximum bid.

### 5.3. Computational Complexity and Convergence Analysis

From analyses above we know the computational complexity of the auction game is *L* in the round of *i*, because the AP will compare *L* coalitions and then onload data from the coalition which maximize its utility in a round. Therefore, the computational complexity of this auction game is LI.

The convergence of this algorithm can be obtained because the maximum point of the utility function in (24) exists, that is to say, a steady Nash equilibrium solution can be found.

**Algorithm 3** LAA-WiFi Second Price Auction Algorithm.1:Define *L* AP coalitions taking part in the auction game with LAA BS through the central controller.2:**Initialization**: AP coalition *i*, $i=\left\{1,2,\ldots ,L\right\}$, sends requests to the LAA BS. After the BS receives the requests and agrees to join the game, the auction begins. $I_{max}=\phi$3:**loop**4: **for** $i=\left\{1,2,\ldots ,L\right\}$ **do**5:  the AP coalition *i* submits its bid $d_{i}$ (compensated slots) and *n* (station numbers waiting for transferring)6: **end for**7: **for** $i=\left\{1,2,\ldots ,L\right\}$ **do**8:  LAA finds the winner satisfying: $I_{max}=\left\{i\in L:i=argmax(u_{LAA})\right\}$9: **end for**10: LAA pays the second-highest payoff to the winner.11:**end loop**


## 6. Simulation Results

In this part, some MATLAB simulations to evaluate the performance of modified CSAT and the proposed two-layer game are presented. The unlicensed spectrums has the bandwidth of 20 MHz and the IEEE 802.11ac protocol working around 5 GHz is used. Other WiFi related simulation parameters are listed in Table 1. The optimal number of stations $n^{*}$ which could maximize the throughput of an AP is 10 by using CVX in MATLAB in the above-mentioned parameter setting environment which has been discussed in Section 2.2 from the calculation results of Equation (7). We assume that there are *K* APs working in the network but we mainly concern one coalition with three APs and one LAA cell which are randomly deployed in the network in 5 GHz unlicensed bands. The fixed station numbers within the coverage of AP1 is 3, which means AP1 is a light-loaded AP. In addition, there are 20 stations in AP3, that is to say, AP3 is a heavy-loaded AP. In addition, the number of stations in AP2 is ranging from 1 to 25 to evaluate the influence on system performance vs. the number of stations changing. Interferences among APs could be ignored due to the short transmission range and low transmission power of the WiFi AP. We further assume the time of a full CSAT cycle is 100 ms and the slot time in the WiFi is 2 ms. The maximum BS transmit power in 5 GHz is 20 dBm and the path loss model in unlicensed band is given as −15.3−50 log10$\left(\hat{d}\left(m\right)\right)$(dB).

**Figure 4:**
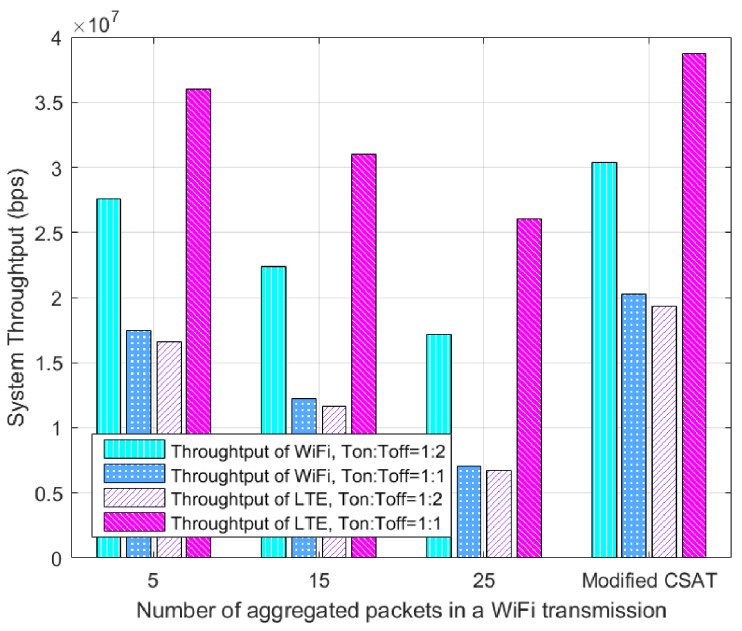
The modified LBT-based CSAT.

Figure 4 reveals the advantages after using the modified CSAT mechanism. The ratio of periodic $T_{on}:T_{off}=1:1$ means the time of the “on” state period and the “off” state period are both 50 ms and the ratio $T_{on}:T_{off}=1:2$ indicates the time for LAA transmission and for WiFi transmission is 33 ms and 67 ms, respectively. Assuming that on average the collision occurs half-way at the start of an LAA transmission [13]. From the figure, we can find that the throughput of both LAA and WiFi decreases with the increased number of aggregated packets [13] in a WiFi transmission due to the increased collision probability and the delay in WiFi. In addition, the trade-off between aggressiveness and fairness of LAA can be adjusted by varying $T_{on}$. In addition, the throughput of LAA is obviously better than that of WiFi and the proposed mechanism can greatly improve the throughput of both LAA and WiFi due to collision avoidance at the instant start of “on” period. For example, when $T_{on}:T_{off}=1:1$ and the number of aggregated packets is 15, the system throughput of WiFi and LAA can increase about 65.93% and 24.76%, respectively, after using the devised approach.

**Figure 5:**
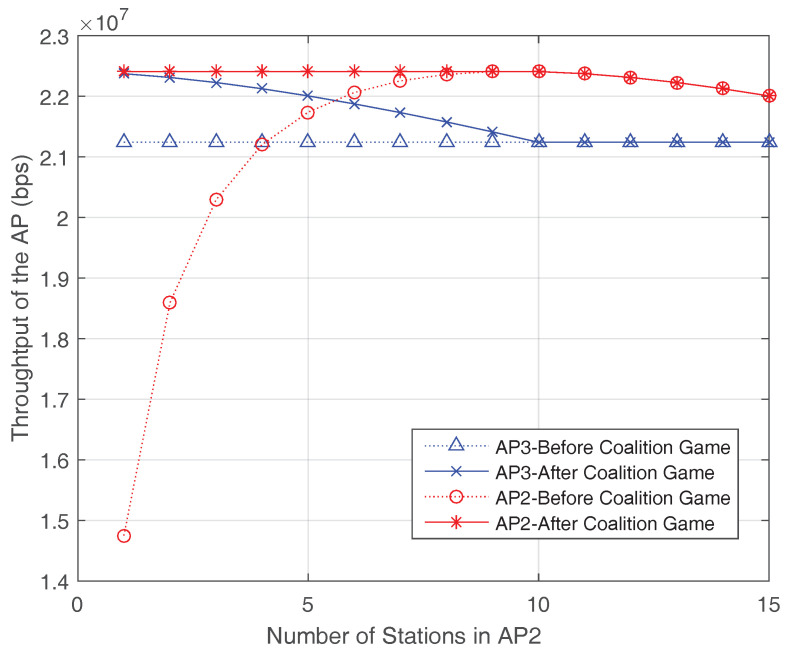
Coalition forming based on merge-and-split rule.

**Figure 6:**
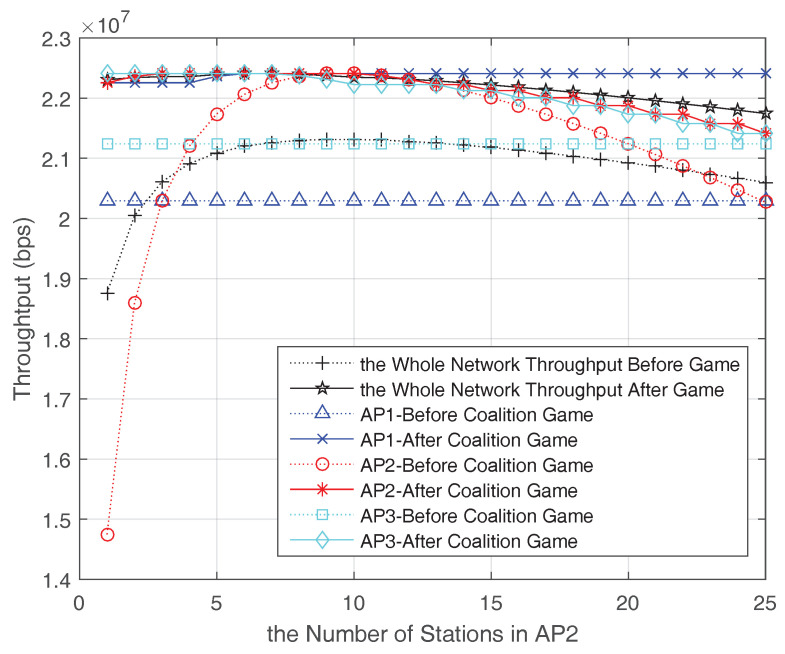
Throughput changes after coalition forming (three APs in the coalition).

Next, the impact of WiFi AP throughput vs. the changing of station numbers before and after coalition forming is presented in Figure 5 and Figure 6. Firstly, we assume that the AP1 is turned off and has no data waiting for transmission. There are only AP2, AP3 and other APs working in the network. The throughput of AP2 and AP3 before and after coalition forming is shown in Figure 5. The AP3 is a heavy-loaded AP, so AP3 has the motivation to form a coalition with other APs which has less stations. When the station number in AP2 is less than $n^{*}$, both APs could benefit from coalition forming based on merged rule. However, when the number of stations in AP2 is above the $n^{*}$, no benefit could get from the coalition anymore and the coalition between AP2 and AP3 would be broken based on the splitting rule. Then, AP2 and AP3 would find other APs for cooperation.

**Figure 7:**
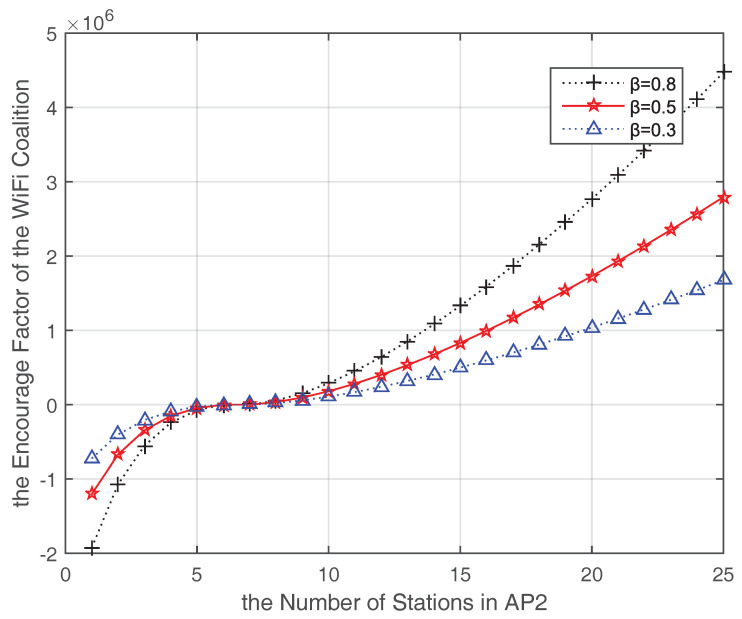
Encourage factor changes after coalition forming (three APs in the coalition).

Moreover, to get insight of the each AP throughput changes after coalition formed, we add the AP1 in the game and the throughput changes of APs before and after coalition forming is shown in Figure 6. As assumed that AP1 is light-loaded and AP3 is heavy-loaded, consequently, after the coalition forming, parts of AP3 data are offloaded to AP1 and AP2 while the number of AP2 stations is less than $n^{*}$. We further assume AP2 and AP3 have the equal channel condition, equal priority and equal distance to AP1, so when *n* is larger than $n^{*}$, AP1 will accept the transferred stations from AP2 and AP3 alternatively based on the Algorithm 1. In addition, with the increasing number of AP2 stations, AP2 and AP3 would share the benefit from AP1’s help. However, once the AP1 station number is over $n^{*}$ (including stations transferred from AP2 and AP3), it would maintain its current station number and does not continue receiving loads any more. At this time, the AP1 throughput could maintain the maximum value while the throughput in AP2 and AP3 would drop due to the increased station number. Even so, AP2 and AP3 could get profits from the coalition because some data are carried by AP1, which can benefit from the coalition to reach the optimal station numbers and maximum throughput. Accordingly, the impact of changing the encourage factor α of the WiFi coalition is shown in Figure 7. β is the discount factor which has effects on the motivation of a AP joining the coalition. A lager β means a stronger motivation. While the average station number is less than $n^{*}$, the encourage factor α is negative, which means more stations could be transferred to this coalition in this period. On the contrary, when the average station number is larger than $n^{*}$, α is positive and increased with the increasing number of stations in AP2. In this case, this coalition is overloaded, if there are other lighted-AP in WiFi network, it could join in this coalition to alleviate the traffic and optimize the WiFi throughput.

**Figure 8:**
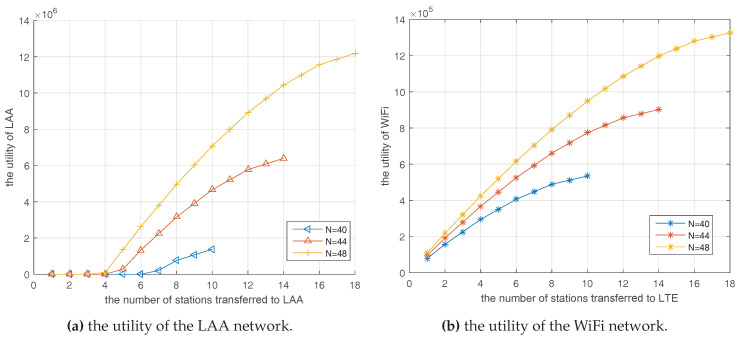
The utility of players in the auction game. (**a**) The utility of the LAA network; (**b**) the utility of the WiFi network.

Figure 8 reveals the utility changes of LAA and WiFi with the number of transferred stations as well as the total number of stations in the AP coalition when using $\theta^{*}$ in Equation (24), respectively. From the observation, once the station number of the AP coalition exceeds the optimal value, this coalition has the motivation to offload some data to the LAA network to optimize its network performance. The more stations transferred to the LAA, the larger utility function may be obtained by the WiFi network. In addition, the compensated slot by the WiFi can provide considerable benefits for the LAA. Furthermore, in the case of transferring the same number of stations, a larger total number of stations in AP coalition, *N*, could lead to more profits after the auction. Because the coalition is more overloaded, it has stronger demands to transfer users to the LAA to reduce collisions, which means LAA could get more rewards from this heavy-loaded AP and would give priority to help this AP. Besides, more stations are transferred to the LAA means more time slots would be compensated for the LAA; consequently, higher throughput can be obtained by using the centralized-control mechanism and better management technology of LTE.

**Figure 9:**
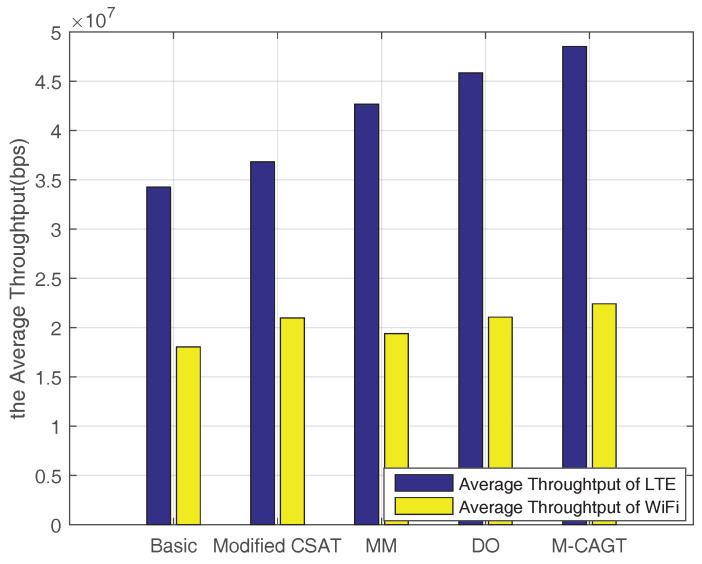
Throughput comparison.

Figure 9 illustrates the network throughput comparison among five schemes. “Basic” means the original LBT-based CSAT mechanism, collisions may occur at the moment of starting the “on” period and “Modified CSAT” indicates the modified mechanism that is discussed in Section 3. “MM” denotes the scheme in [19] using a Min-Max Fairness Algorithm to make a traffic transaction using a coalition game (15% of macrocell throughput is offloaded to WiFi APs). “DO” indicates the scheme in [16] which offloads parts of data from WiFi to LAA based on the distance and channel condition. In addition, M-CAGT means the CAGT scheme with modified CSAT used in this paper. In terms of the average total throughput, the proposed Coalition-Auction scheme based on modified CSAT can achieve about 41.7%, 31.8%, 13.74% and 5.9% improvement for LAA compared to baseline CSAT, modified CSAT, “MM” and “DO”, respectively. In addition, the scheme in this paper can obtain about 24.2%, 6.85%, 15.51% and 6.4% promotion for WiFi compared to baseline CSAT, modified CSAT, the scheme in [19] and the scheme in [16], respectively.

## 7. Conclusions

In this paper, we investigate the coexistence between LAA and WiFi systems in unlicensed bands. Especially, we propose a modified CSAT mechanism, which could avoid the collision at the start of CSAT “on” moment. In addition, a two-layer CATG scheme is proposed which could optimize the performance of both LAA and WiFi system simultaneously. Simulation results show that the proposed scheme improves the whole network’s throughput effectively. We expect that the proposed scheme is a promising approach for enhancing the performance of coexisting networks.

## Figures and Tables

**Table 1:** WiFi system paramaters.

Parameters	Value	Parameters	Value
slot time	9 μs	Unlicensed bandwidth	20 MHz
PHY header of WiFi packet	224 bits	Station transmission probability τ	0.05
PHY header of WiFi packet	224 bits	AWGN noise power	−174 dBm/Hz
SIFS	16 μs	WiFi slot time size, σ	20 μs
DIFS	34 μs	ACK	112 bits + 224 bits
WiFi maximum backoff state $m^{\left(WF\right)}$	6	Channel bit rate for WiFi	300 Mbps

## Appendix A. Derivation of Transition Probability of Markov Chain

For $AP_{x}$, the transition probability $P_{x}\left(x\in S_{L}\in Y\to x\in S_{L}^{\prime }\in Y^{\prime }\right)$ in Equation (14) means the probability that $AP_{x}$ changes from coalition $S_{L}\in Y$ to coalition $S_{L}^{\prime }\in Y^{\prime }$. Because APs form coalitions based on the Transfer Rule and Merge-and-Split Rule, both of which depend on the utility function of the player, the throughput changes of $AP_{x}$ can clearly show the utility function variation.

We assume $AP_{x}$, which has $n_{x}$ stations, is a lighted-loaded AP which will find overloaded APs to form coalitions. $n_{l,a}$ and $n_{l,a}^{\prime }$ mean the number of stations waiting to be offloaded from heavy-loaded APs in coalition $S_{L}$ and $S_{L}^{\prime }$, respectively. $n_{l,s}$ and $n_{l,s}^{\prime }$ indicate the number of stations which could handover to the light-loaded APs in coalition $S_{L}$ and $S_{L}^{\prime }$. In addition, $n^{*}$ is the optimal station number which could maximize the $AP_{x}$ throughput. Accordingly, $▵=\sum_{S}n_{l,a}-\sum_{S}n_{l,s}$ and ${▵}^{\prime }=\sum_{S^{\prime }}n_{l,a}^{\prime }-\sum_{S^{\prime }}n_{l,s}^{\prime }$ mean the station number which are expected to be offloaded to $AP_{x}$ after $AP_{x}$ joining in coalition $S_{L}$ and $S_{L}^{\prime }$, respectively. Next, we consider four cases for the derivation of transition probability.

Case 1: $▵\geq n_{x}$ and ${▵}^{\prime }\geq n_{x}$.

(A1) $$\begin{array}{cc} & P_{x}\left(x\in S_{L}\in Y\to x\in S_{L}^{\prime }\in Y^{\prime }\right)\\ = & P_{x}\left\{\nu_{x}\left(S_{L}^{\prime }\right)\geq \nu_{x}\left(S_{L}\right)\right\}=P_{x}\left\{R_{x}\left(n^{*}\right)\geq R_{x}\left(n^{*}\right)\right\}=1. \end{array}$$

Case 2: $▵\geq n_{x}$ and ${▵}^{\prime }<n_{x}$.

(A2) $$\begin{array}{cc} & P_{x}\left(x\in S_{L}\in Y\to x\in S_{L}^{\prime }\in Y^{\prime }\right)\\ = & P_{x}\left\{\nu_{x}\left(S_{L}^{\prime }\right)\geq \nu_{x}\left(S_{L}\right)\right\}=P_{x}\left\{R_{x}\left(n^{*}\right)\geq R_{x}\left(n_{x}+▵\right)\right\}\\ = & P_{x}\{\frac{an^{*}\tau {\left(1-\tau \right)}^{n^{*}-1}E_{x}\left[P\right]}{\left(T_{\sigma }-T_{c}\right){\left(1-\tau \right)}^{n^{*}}+\left(T_{S}-T_{c}\right)n^{*}\tau {\left(1-\tau \right)}^{n^{*}-1}+T_{c}}\\ & \geq \frac{a\left(n_{x}+▵\right)\tau {\left(1-\tau \right)}^{n_{x}+▵-1}E_{x}\left[P\right]}{\left(T_{\sigma }-T_{c}\right){\left(1-\tau \right)}^{\left(n_{x}+▵\right)}+\left(T_{S}-T_{c}\right)\left(n_{x}+▵\right)\tau {\left(1-\tau \right)}^{(n_{x}+▵-1)}+T_{c}}\}. \end{array}$$

Case 3: $▵<n_{x}$ and ${▵}^{\prime }\geq n_{x}$.

(A3) $$\begin{array}{cc} & P_{x}\left(x\in S_{L}\in Y\to x\in S_{L}^{\prime }\in Y^{\prime }\right)\\ = & P_{x}\left\{\nu_{x}\left(S_{L}^{\prime }\right)\geq \nu_{x}\left(S_{L}\right)\right\}=P_{x}\left\{R_{x}\left(n_{x}+{▵}^{\prime }\right)\geq R_{x}\left(n^{*}\right)\right\}\\ = & P_{x}\{\frac{a\left(n_{x}+{▵}^{\prime }\right)\tau {\left(1-\tau \right)}^{n_{x}+{▵}^{\prime }-1}E_{x}\left[P\right]}{\left(T_{\sigma }-T_{c}\right){\left(1-\tau \right)}^{\left(n_{x}+{▵}^{\prime }\right)}+\left(T_{S}-T_{c}\right)\left(n_{x}+{▵}^{\prime }\right)\tau {\left(1-\tau \right)}^{\left(n_{x}+{▵}^{\prime }-1\right)}+T_{c}}\\ & \geq \frac{an^{*}\tau {\left(1-\tau \right)}^{n^{*}-1}E_{x}\left[P\right]}{\left(T_{\sigma }-T_{c}\right){\left(1-\tau \right)}^{n^{*}}+\left(T_{S}-T_{c}\right)n^{*}\tau {\left(1-\tau \right)}^{n^{*}-1}+T_{c}}\}. \end{array}$$

Case 4: $▵<n_{x}$ and ${▵}^{\prime }<n_{x}$.

(A4) $$\begin{array}{cc} & P_{x}\left(x\in S_{L}\in Y\to x\in S_{L}^{\prime }\in Y^{\prime }\right)\\ = & P_{x}\left\{\nu_{x}\left(S_{L}^{\prime }\right)\geq \nu_{x}\left(S_{L}\right)\right\}=P_{x}\left\{R_{x}\left(n_{x}+{▵}^{\prime }\right)\geq R_{x}\left(n_{x}+▵\right)\right\}\\ = & P_{x}\{\frac{a\left(n_{x}+{▵}^{\prime }\right)\tau {\left(1-\tau \right)}^{n_{x}+{▵}^{\prime }-1}E_{x}\left[P\right]}{\left(T_{\sigma }-T_{c}\right){\left(1-\tau \right)}^{\left(n_{x}+{▵}^{\prime }\right)}+\left(T_{S}-T_{c}\right)\left(n_{x}+{▵}^{\prime }\right)\tau {\left(1-\tau \right)}^{\left(n_{x}+{▵}^{\prime }-1\right)}+T_{c}}\\ & \geq \frac{a\left(n_{x}+▵\right)\tau {\left(1-\tau \right)}^{n_{x}+▵-1}E_{x}\left[P\right]}{\left(T_{\sigma }-T_{c}\right){\left(1-\tau \right)}^{\left(n_{x}+▵\right)}+\left(T_{S}-T_{c}\right)\left(n_{x}+▵\right)\tau {\left(1-\tau \right)}^{\left(n_{x}+▵-1\right)}+T_{c}}\}. \end{array}$$

In Case 4, if ${▵}^{\prime }<▵$, the $\varphi_{x}\left(Y^{\prime }|Y\right)$ of $AP_{x}$ in = Equation (15) will be equal to zero because leaving from coalition $S_{L}$ and joining coalition $S_{L}^{\prime }$ cannot bring more benefits to $AP_{x}$. The similar result would be happened in Case 2. Thus, the probability of $AP_{x}$ transmitting from $S_{L}\in Y$ to coalition $S_{L}^{\prime }\in Y^{\prime }$ is zero in these two cases. In Case 4, if ${▵}^{\prime }\geq ▵$, the player $AP_{x}$ can get more benefits to join in coalition $S_{L}^{\prime }$. Thus, transmitting probability of $AP_{x}$ from $S_{L}\in Y$ to $S_{L}^{\prime }\in Y^{\prime }$ is positive. In addition, in Case 1, staying in coalition $S_{L}$ or joining in coalition $S_{L}^{\prime }$ is comparative because the utility function of $AP_{x}$ is equal in these two coalitions.
